# Prevalence of RAS and BRAF mutations in metastatic colorectal cancer patients by tumor sidedness: A systematic review and meta‐analysis

**DOI:** 10.1002/cam4.2747

**Published:** 2019-12-19

**Authors:** Lauren C. Bylsma, Christina Gillezeau, Tamer A. Garawin, Michael A. Kelsh, Jon P. Fryzek, Laura Sangaré, Kimberly A. Lowe

**Affiliations:** ^1^ EpidStat Institute Ann Arbor MI USA; ^2^ Amgen Thousand Oaks CA USA; ^3^ SimulStat Portland OR USA; ^4^ Amgen Seattle WA USA

**Keywords:** BRAF, KRAS, metastatic colorectal cancer, RAS, tumor sidedness

## Abstract

Studies have shown that the prevalence of RAS and BRAF mutations may differ by tumor sidedness among metastatic colorectal cancer (mCRC) patients. Both mutation status and tumor sidedness may impact survival and disease progression and RAS mutation status has been shown to predict response to anti‐epidermal growth factor receptor (EGFR) therapy. A systematic literature review and meta‐analysis were conducted to estimate the pooled prevalence of RAS and BRAF mutations by tumor sidedness in studies of mCRC patients. Forty‐four studies comprising 15 981 mCRC patients tested for RAS and/or BRAF mutations were included in the meta‐analyses. The prevalence of RAS mutations differed significantly by tumor side (32.4% among left‐sided tumors, 41.3% among right‐sided tumors; *P* = .017), as did the prevalence of KRAS mutations (35.8% among left‐sided tumors, 46.3% among right‐sided tumors; *P* < .0001) and BRAF mutations (4.3% among left‐sided tumors, 16.3% among right‐sided tumors; *P* < .0001). Among right‐sided tumors, the prevalence of RAS and KRAS mutations varied significantly by study design, with higher prevalence among observational studies than clinical trials, and there was significant variation by study location for the prevalence of KRAS mutations in left‐sided tumors and the prevalence of BRAF mutations in right‐sided tumors. These results help to better characterize the mCRC population to better inform clinicians and researchers. Few of the included studies reported overall or progression‐free survival (PFS) by both tumor sidedness and mutation status. As both of these factors may have prognostic impact, future studies should consider evaluating survival by these variables.

## Graphical abstract

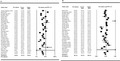

A systematic literature review and meta‐analysis were conducted to estimate the prevalence of RAS and BRAF mutations by tumor sidedness in metastatic colorectal cancer (mCRC) patients. RAS, KRAS, and BRAF mutations were found to vary significantly by tumor location, occurring more frequently among right‐sided colon tumors than left‐sided colon tumors. These results inform clinicians and researchers regarding the mCRC patient population and potential targeted patients for anti‐EGFR therapies.

## 1 INTRODUCTION

Approximately 20% of new colorectal cancer (CRC) cases are metastatic (mCRC) at diagnosis and another 20% of cases progress to metastatic disease.^1^, ^2^ RAS mutations have been associated with a lack of response to anti‐epidermal growth factor receptor (EGFR) monoclonal antibody therapies used in mCRC treatment such as cetuximab and panitumumab.^3^, ^4^ The American Society for Clinical Oncology recommends that all mCRC patients who are candidates for anti‐EGFR therapies should first be tested for the Kirsten RAS (KRAS) mutation.^4^ The requirement to establish RAS status prior to administration of an EGFR has increased the need for more information on the epidemiologic and tumor characteristics by RAS status. A recent pooled analysis of randomized controlled trials of mCRC patients reported significant differences in RAS mutation prevalence estimates by clinical trial, sex, and by country.^5^

There is evidence to suggest that tumor biology and pathology differ for right‐ and left‐sided colon tumors.^6^, ^7^, ^8^, ^9^ The two sides of the colon have differing developmental origins whereby the right colon originates from the embryonic midgut and the left colon originates from the hindgut.^10^ Anatomically, the two sides have distinct lymphatic systems and lumen environments, and blood is supplied by different arteries. Risk factors for colon cancer have been shown to vary by side as well with right‐sided colon cancer being associated with female sex, increasing age, history of cancer, and insulin resistance, while left‐sided tumors have been associated with heavy smoking, alcohol abuse, and a low fiber diet.^11^ A survey of pathology centers across Europe observed that the prevalence of RAS mutations was higher in right‐sided tumors than left‐sided tumors in these centers (54.6% vs 46.4%, respectively).^12^ Patients with right‐sided colon tumors have been shown to have worse prognosis and it has been hypothesized that genetic differences in the tumor may account for these findings.^8^, ^9^, ^13^, ^14^ A recent meta‐analysis found that left‐sided colon tumors had a significantly reduced risk of mortality compared with right‐sided colon tumors (hazard ratio [HR]: 0.82; 95% confidence interval [CI]: 0.79‐0.84) among 1.5 million pooled patients.^15^ In addition, progression‐free survival (PFS) has been shown to be significantly improved among patients with wild‐type KRAS left‐sided tumors who were treated with cetuximab compared to best supportive care (median survival was 5.4 months vs 1.8 months, respectively).^6^

The b‐type Raf proto‐oncogene (BRAF) mutation is a novel biomarker that is gaining interest due to its association with a worse prognosis when compared to BRAF wild‐type CRCs.^16^, ^17^ A recent meta‐analysis found that CRC patients with BRAF mutations had worse overall survival (OS) and PFS on anti‐EGFR therapies compared to patients with wild‐type BRAF cancer.^18^ Some studies have suggested that BRAF mutations may occur more frequently in right‐sided colon cancers than in left‐sided cancers.^11^, ^17^, ^19^ The majority of BRAF‐activating mutations occur in codon 600 (V600E).^20^ These mutations occur less frequently in nonhigh microsatellite instability (MSI‐H) tumors, where they confer a strong negative prognostic marker for CRC patients.^20^, ^21^ However, among MSI‐H tumors, where the mutation more frequently occurs, the V600E mutation does not have the same adverse prognostic value.^20^, ^21^ Data on BRAF mutations occurring outside of codon 600 are sparse. Data from one retrospective cohort study suggest that non‐V600E BRAF mutations occur in younger individuals (58 years vs 69 years), and less frequently occur in females (46% vs 65%), are less often high grade (13% vs 64%) and on the right side (36% vs 81%) compared to patients with V600E mutations.^22^ The median OS of non‐V600E BRAF mutations was longer relative to those with V600E mutations.^22^

To our knowledge, no robust summary of the prevalence of RAS and BRAF mutations by tumor sidedness in mCRC patients has been published. Thus, we conducted a systematic review and meta‐analysis of the available scientific literature. A meta‐analysis of OS and PFS by mutation status and tumor location was a secondary objective among the included studies reporting these outcomes.

## 2 MATERIALS AND METHODS

### 2.1 Literature search and inclusion criteria

This study was conducted in accordance with PRISMA guidelines.^23^ A systematic literature review was conducted in PubMed, Embase, Web of Science, and Cochrane Central Register of Controlled Trials in September 2017 for studies published in the English language since 1 January 2006. The search string included the following terms: (RAS, KRAS, NRAS and/or BRAF) and (mCRC), with term expansion using the MeSH thesaurus to ensure there were no gaps in the search language. We included studies with information on the prevalence of KRAS, NRAS, and BRAF mutations by tumor sidedness among patients with mCRC. Studies with overall and/or PFS outcomes by mutation status and tumor location were included if available, but these outcomes were not part of the specified inclusion criteria. Observational study designs (cohort, case‐control, and case series of 20 or more patients) and standard‐of‐care arms from clinical trials were included. We excluded studies of nonhuman populations, pediatric populations, early stage CRC only, opinion pieces, case series with <20 patients, and articles without original data. We also excluded studies that selected patients based on mutation status or tumor sidedness. The bibliographies of systematic reviews identified in the search were screened for relevant references.

### 2.2 Data extraction and study quality

Two reviewers (LCB, CG) screened the studies at the level of title and abstract and then full‐text. Disagreements over inclusion were resolved by consensus adjudication. Studies were extracted into a standardized extraction database. Extracted variables included study characteristics, population demographics, disease characteristics, primary tumor location, mutation characteristics, mutation assessment method, median OS data, and median PFS data, if reported. Two reviewers (LCB, CG) independently scored each study and all differences were resolved by discussion until consensus was reached. If the original study unspecified tumor side as “left” or “right,” we defined descending, sigmoid, or distal tumors as left‐sided colon tumors. Right‐sided tumors included ascending, transverse, and proximal tumors. During the process of data extraction, several studies were identified with data on mutation status and tumor sidedness that was either not reported for metastatic or stage IV CRC specifically or was not reported for both mutation status and tumor sidedness combined. The authors of these studies were contacted in order to obtain the relevant prevalence data.

Risk of bias in individual studies was assessed using the Strengthening the Reporting of Observational Studies in Epidemiology (STROBE) checklist.^24^ Use of this instrument to assess risk of bias is common in systematic reviews and meta‐analyses.^25^, ^26^, ^27^ The checklist includes 22 items recommended to be reported in observational studies, including description of study design, statistical analysis, potential biases, pertinent results, limitations, and study generalizability. Studies were given one point per checklist item if they sufficiently included the criteria. To assess the potential impact of risk of bias in individual studies on mutation prevalence by tumor side, subgroup analyses were conducted for studies with STROBE scores above and below the median score. A meta‐regression on continuous STROBE score was also conducted.

### 2.3 Statistical analysis

Random‐effects models were used to calculate pooled estimates for the prevalence of KRAS and BRAF mutations by right‐ and left‐sided colon location along with 95% CIs. Studies were weighted using the DerSimonian and Laird methods.^28^ Heterogeneity between studies and subgroups in each analysis was evaluated using the Cochran's *Q* test. Forest plots were generated using the Comprehensive Meta‐Analysis software (version 3.0; Biostat). Publication bias was assessed visually with funnel plots and statistically using Egger's regression method. In analyses with statistically significant publication bias, the Duval and Tweedie trim and fill method was used to estimate the adjusted pooled prevalence after imputing studies that were “missing” in asymmetrical funnel plots.^29^ As some included studies categorized transverse colon cancers separately from right‐sided tumors, sensitivity analyses were performed to evaluate the prevalence of mutations among right‐sided tumors specifically excluding the transverse colon, and among transverse colon cancers alone. Additionally, though “left‐sided” colon cancers typically included rectal cancers, some studies categorized rectal cancers separately from left‐sided colon cancers. Thus, sensitivity analyses were conducted stratified by left‐sided tumors specifically excluding rectal tumors and by rectal tumors alone. Stratified subgroup analyses were conducted for factors selected a priori to potentially impact the prevalence of these mutations, including study country, site of metastasis, source of tissue, study design, dates that the study was conducted, median age of participants, mutation assessment method, median length of follow‐up time, and study quality score, and were limited to analyses including at least three studies. The categories within certain subgroups (median age, study dates, follow‐up time) were chosen based on the distribution observed in the included studies. If the included studies reported OS or PFS outcomes by tumor sidedness and mutation status, these data were extracted. Exploratory meta‐analyses were conducted for studies reporting HRs evaluating the prognostic impact of tumor sidedness regardless of location.

## 3 RESULTS

**Figure 1:**
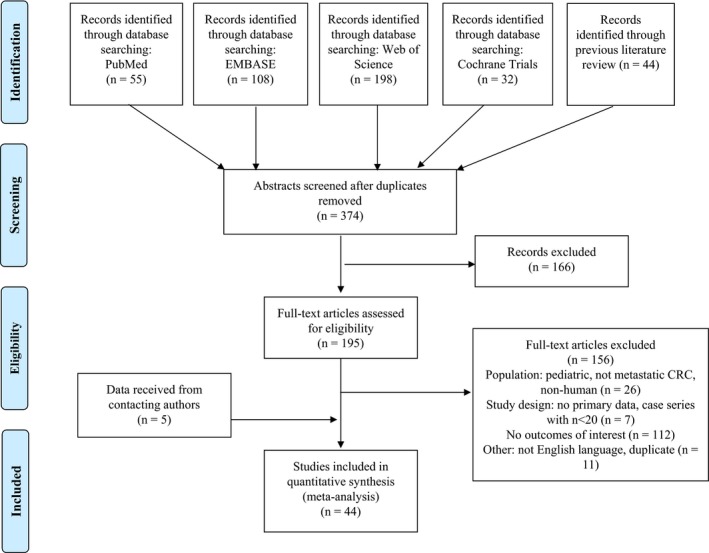
Flow diagram for study inclusion

The flow diagram for study inclusion is presented in Figure 1. After removing duplicates, the search yielded 374 potentially relevant abstracts of which a full‐text review was conducted for 195 studies. Ultimately, 39 studies met the inclusion criteria. An additional 65 studies contained relevant data for KRAS, NRAS, or BRAF mutation status and tumor sidedness but did not report data specific to the mCRC population or data for mutation status by tumor sidedness. We contacted the authors of these studies for data availability and received relevant data from five studies.^30^, ^31^, ^32^, ^33^, ^34^ A total of 44 studies^6^, ^7^, ^19^, ^30^, ^31^, ^32^, ^33^, ^34^, ^35^, ^36^, ^37^, ^38^, ^39^, ^40^, ^41^, ^42^, ^43^, ^44^, ^45^, ^46^, ^47^, ^48^, ^49^, ^50^, ^51^, ^52^, ^53^, ^54^, ^55^, ^56^, ^57^, ^58^, ^59^, ^60^, ^61^, ^62^, ^63^, ^64^, ^65^, ^66^, ^67^, ^68^, ^69^, ^70^ were therefore included in the narrative review and meta‐analyses. The characteristics of the included studies are presented in Table S1.

The 44 included studies comprised 15 981 mCRC patients tested for RAS and/or BRAF mutations. Most of the studies (n = 38) were observational (30 retrospective, eight prospective) and six were from randomized controlled trials. Seventeen studies were conducted in Europe, 11 in Asia, nine in the USA, three in Australia, and four were conducted in multiple countries. The proportion of males in each study ranged from 37.2% to 71.90%, and the mean age ranged from 54.6 to 75.8 years. A variety of technologies were used for mutation assessment, including direct sequencing, PCR, Sequenom, pyrosequencing, Sanger sequencing, Luminex, BigDye Terminator, and VE1 immunohistochemistry. The STROBE checklist score ranged from 8 to 21, with a median score of 16. The KRAS mutation prevalence was assessed in 30 studies, BRAF was assessed in 27 studies, and NRAS was evaluated in three studies. Four studies evaluated overall RAS mutations only.

**Figure 2:**
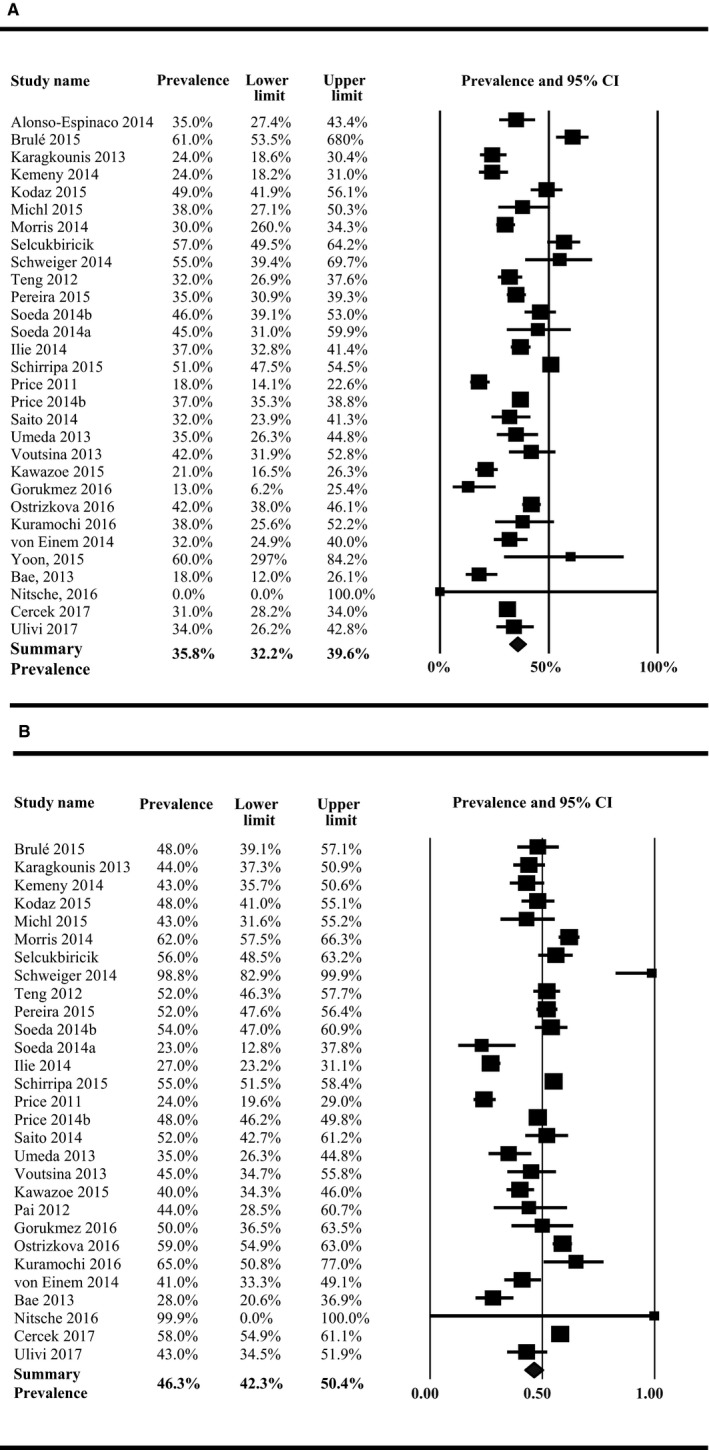
Prevalence of all KRAS mutations among (A) left‐sided colon cancers, (B) right‐sided colon cancers

**Figure 3:**
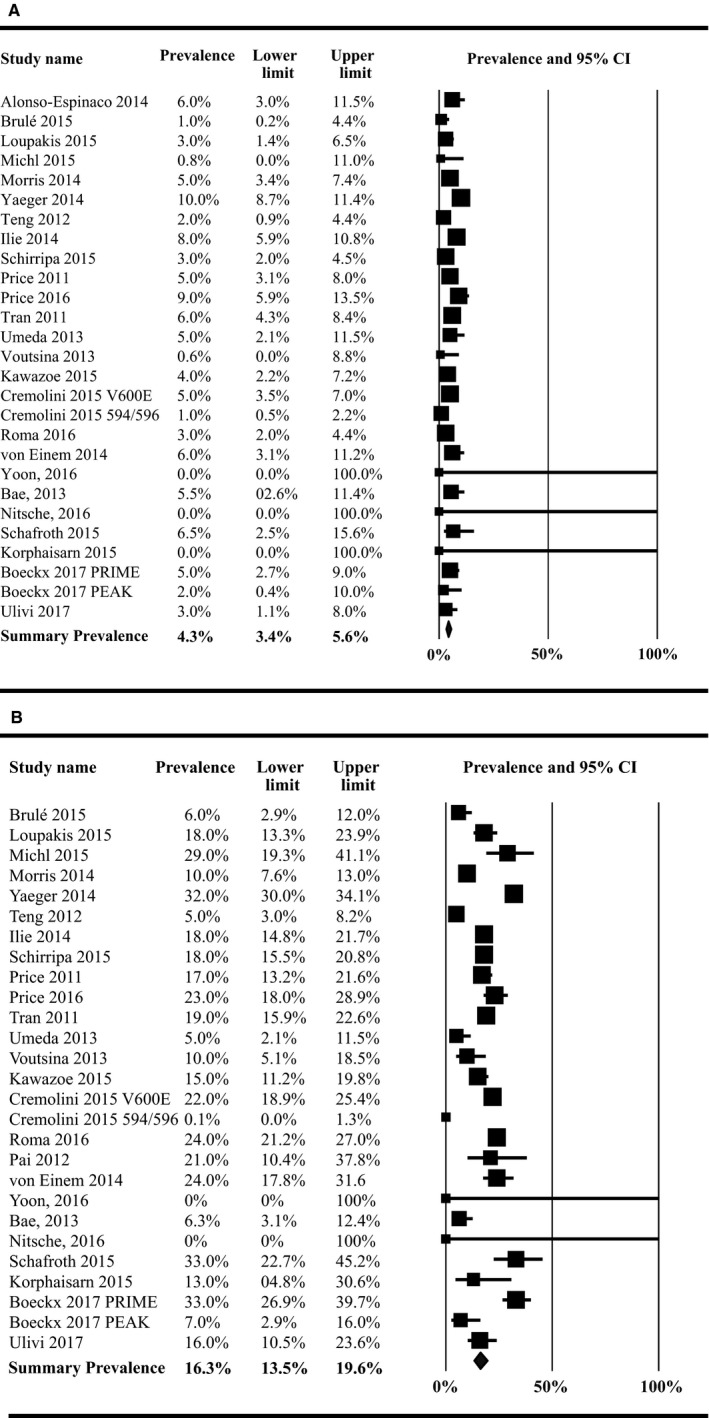
Prevalence of all BRAF mutations among (A) left‐sided colon cancers, (B) right‐sided colon cancers

The prevalence of all RAS mutations was found to vary significantly by tumor location (Cochran's *Q* *P* = .017), with higher prevalence among right‐sided colon tumors (41.3%, 95% CI: 35.4%‐47.5%) than left‐sided colon tumors (32.4%, 95% CI: 28.4%‐36.7%) (Table 1). The prevalence of NRAS did not differ significantly by location (*P* = .931) (right‐sided tumors: 6.5%, 95% CI: 5.5%‐7.7%; left‐sided tumors: 6.2%, 95% CI: 1.5%‐21.7%), although this mutation was only evaluated in three studies. KRAS mutations were found to vary significantly by tumor side (*P* < .0001), with 46.3% (95% CI: 42.3%‐50.4%) of right‐sided colon cancers harboring a KRAS mutation compared to 35.8% (95% CI: 32.2%‐39.6%) of left‐sided tumors (Figure 2A,B). Some studies reported prevalence estimates of 0% (no mutations identified) or 100% (all samples had mutations); the prevalence estimates from these studies had wide CI in the meta‐analyses due to the large variance. The prevalence of BRAF mutations was 16.3% (95% CI: 13.5%‐19.6%) among right‐sided colon tumors and 4.3% (95% CI: 3.4%‐5.6%) among left‐sided colon tumors (*P* < .0001) (Figure 3A,B). Each of the overall prevalence analyses had statistically significant heterogeneity present, with the exception of NRAS mutation among right‐sided tumors.

**Table 1:** Overall prevalence of RAS, KRAS, and BRAF mutations by primary tumor location

	RAS mutation		KRAS mutation		BRAF mutation		NRAS mutation	
	Left‐sided tumors	Right‐sided tumors	Left‐sided tumors	Right‐sided tumors	Left‐sided tumors	Right‐sided tumors	Left‐sided tumors	Right‐sided tumors
Mutation prevalence (95% CI)	32.4% (28.4%‐36.7%)	41.3% (35.4%‐47.5%)	35.8% (32.2%‐39.6%)	46.3% (42.3%‐50.4%)	4.3% (3.4%‐5.6%)	16.3% (13.5%‐19.6%)	6.2% (1.5%‐21.7%)	6.5% (5.5%‐7.7%)
N studies included	38	37	30	29	27	27	3	3
*P*‐value for heterogeneity; *I* ^2^ value	<.0001; 95.4%	<.0001; 97.4%	<.0001; 91.0%	<.0001; 91.7%	<.0001; 80.7%	<.0001; 91.7%	<.0001; 97.8%	.68; 0.00%
Cochran's *Q* test for heterogeneity between groups	*P* = .17		*P* < .0001		*P* < .0001		*P* = .931	

Subgroup analyses evaluating variations in mutation prevalence by side for a priori study characteristics are shown in Table 2. The prevalence of RAS mutations differed significantly by study design with higher prevalence for observational studies than randomized controlled trial (RCTs) among right‐sided tumors (*P* = .040), and studies with a STROBE score of <16 reported a higher prevalence of RAS mutations among both left‐ and right‐sided tumors, when compared with studies with STROBE scores of 16 or higher. However, the meta‐regression by continuous STROBE score did not demonstrate a significant effect by this factor. The prevalence of KRAS mutations was statistically significantly different by study country in left‐sided colon cancers (*P* = .003) and significantly different by study design among right‐sided tumors (*P* = .035). The prevalence of BRAF mutations was statistically significantly different by mutation assessment method in left‐sided cancers (*P* = .001) and among study country (*P* = .041) and median length of follow‐up (*P* = .007) among right‐sided cancers.

**Table 2:** Subgroup analyses of RAS, KRAS, and BRAF mutation prevalence by primary tumor location

	RAS mutation prevalence (95% CI) (n studies)		KRAS mutation prevalence (95% CI) (n studies)		BRAF mutation prevalence (95% CI) (n studies)	
	Left‐sided tumors	Right‐sided tumors	Left‐sided tumors	Right‐sided tumors	Left‐sided tumors	Right‐sided tumors
Study location						
US	25.6% (19.8%‐32.5%) (n = 9)	38.1% (25.0%‐53.1%) (n = 10)	29.6% (26.2%‐33.2%) (n = 5)	51.7% (45.6%‐57.8%) (n = 6)	a	19.4% (7.3%‐42.3%) (n = 3)
EU	32.7% (23.5%‐43.3%) (n = 13)	38.6% (25.4%‐53.8%) (n = 12)	40.3% (35.2%‐45.6%) (n = 10)	47.0% (36.5%‐57.8%) (n = 9)	3.8% (2.6%‐5.6%) (n = 12)	20.5% (17.1%‐24.4%) (n = 11)
Other	36.1% (30.2%‐42.5%) (n = 16)	46.1% (40.4%‐51.9%) (n = 15)	35.8% (29.5%‐42.6%) (n = 15)	44.3% (38.9%‐49.8%) (n = 14)	4.6% (3.5%‐6.0%) (n = 13)	13.0% (9.4%‐17.6%) (n = 13)
Cochran's *Q*	*P* = .076	*P* = .444	***P* = .003**	*P* = .209	*P* = .280	***P* = .041**
Metastasis site						
Liver	30.0% (25.7%‐34.8%) (n = 6)	48.2% (41.4%‐55.0%) (n = 6)	29.5% (24.7%‐34.9%) (n = 5)	46.9% (39.5%‐54.4%) (n = 5)	a	a
Lung	a	a	a	a	a	a
Tissue source						
Primary tumor	33.0% (20.4%‐48.7%) (n = 7)	44.2% (21.6%‐59.5%) (n = 6)	40.4% (32.5%‐48.9%) (n = 6)	53% (41.2%‐64.5%) (n = 5)	4.2% (2.7%‐6.6%) (n = 5)	15.5% (9.2%‐24.7%) (n = 4)
Metastasis	a	a	a	a	a	a
Study design						
Observational	32.6% (28.3%‐37.1%) (n = 32)	44.0% (37.4%‐50.8%) (n = 31)	35.7% (32.1%‐39.5%) (n = 25)	48.6% (44.6%‐52.7%) (n = 24)	4.5% (3.3%‐6.0%) (n = 21)	16.2% (13.0%‐20.1%) (n = 21)
Clinical trial	28.4% (15.5%‐46.2%) (n = 6)	28.7% (18.5%‐41.7%) (n = 6)	36.7% (21.6%‐55.0%) (n = 5)	35.6% (25.7%‐47.0%) (n = 5)	4.3% (2.9%‐6.2%) (n = 6)	16.2% (10.3%‐24.7%) (n = 6)
*P*‐value	*P* = .626	***P* = .040**	*P* = .909	***P* = .035**	*P* = .850	*P* = .995
Study dates						
Pre‐2007	35.5% (15.4%‐62.6%) (n = 3)	38.8% (28.4%‐50.4%) (n = 3)	35.5% (15.4%‐62.6%) (n = 3)	38.8% (28.4%‐50.4%) (n = 3)	4.7% (2.6%‐8.4%) (n = 4)	14.4% (6.2%‐30.0%) (n = 4)
Includes 2007	32.0% (26.3%‐38.3%) (n = 21)	40.6% (32.9%‐48.8%) (n = 21)	36.0% (31.1%‐41.1%) (n = 18)	46.5% (39.9%‐53.2%) (n = 18)	4.4% (3.2%‐6.0%) (n = 15)	14.6% (11.4%‐18.4%) (n = 16)
Post‐2007	31.2% (22.8%‐41.1%) (n = 10)	41.0% (26.3%‐57.6%) (n = 10)	38.1% (28.5%‐58.8%) (n = 6)	49.4% (41.9%‐57.0%) (n = 6)	5.1% (2.0%‐12.1%) (n = 3)	21.0% (12.3%‐33.6%) (n = 3)
Cochran's *Q*	*P* = .949	*P* = .962	*P* = .932	*P* = .319	*P* = .949	*P* = .451
Median age						
<63 (<65 for BRAF)	28.5% (23.2%‐34.4%) (n = 16)	40.8% (30.0%‐52.6%) (n = 15)	32.1% (27.4%‐37.3%) (n = 13)	50.0% (45.0%‐54.9%) (n = 12)	4.2% (2.8%‐6.3%) (n = 14)	13.7% (9.2%‐20.0%) (n = 11)
≥63 (≥65 for BRAF)	31.8% (23.9%‐40.9%) (n = 14)	36.1% (26.6%‐46.9%) (n = 14)	39.1% (32.0%‐46.6%) (n = 8)	41.6% (34.1%‐49.6%) (n = 11)	5.2% (3.4%‐7.7%) (n = 6)	18.7% (15.5%‐22.4%) (n = 8)
Cochran's *Q*	*P* = .524	*P* = .552	*P* = .120	*P* = .080	*P* = .494	*P* = .149
Meta‐regression on median age	0.05 (−0.01 to 0.11) (n = 30)	−0.01 (−0.08 to 0.06) (n = 29)	0.02 (−0.03 to 0.07) (n = 24)	−0.04 (−0.08 to 0.003) (n = 23)	0.02 (−0.04 to 0.09) (n = 20)	0.05 (−0.02 to 0.34) (n = 19)
Mutation assessment method						
Sanger sequencing/pyrosequencing	35.2% (30.7%‐40.0%) (n = 8)	40.6% (30.7%‐51.4%) (n = 8)	34.8% (31.9%‐37.9%) (n = 6)	44.5% (33.2%‐56.3%) (n = 6)	7.9% (5.1%‐12.1%) (n = 4)	20.4% (13.3%‐30.0%) (n = 5)
PCR or direct sequencing	32.5% (24.2%‐42.1%) (n = 14)	43.9% (31.8%‐56.8%) (n = 13)	34.3% (27.2%‐42.2%) (n = 11)	46.9% (36.2%‐57.9%) (n = 10)	4.5% (3.2%‐6.2%) (n = 8)	13.0% (8.9%‐18.6%) (n = 7)
BigDye Terminator	20.2% (6.1%‐49.5%) (n = 4)	36.7% (15.6%‐64.4%) (n = 5)	31.8% (17.5%‐50.6%) (n = 3)	53.7% (50.8%‐56.6%) (n = 4)	2.9% (2.2%‐3.7%) (n = 3)	15.3% (9.5%‐23.7%) (n = 4)
Other	31.0% (19.5%‐45.3%) (n = 4)	38.0% (12.3%‐73.0%) (n = 4)	a	a	a	a
Cochran's *Q*	*P* = .656	*P* = .955	*P* = .983	*P* = .254	***P* = .001**	*P* = .352
Mutation assessment method						
Mass spectrometry	29.9% (23.1%‐37.8%) (n = 5)	40.1% (22.1%‐61.4%) (n = 5)	35.4% (28.2%‐43.4%) (n = 3)	54.1% (41.2%‐66.4%) (n = 3)	3.2% (1.0%‐9.1%) (n = 3)	13.4% (6.1%‐26.9%) (n = 3)
Multiplex mutation assay	31.2% (19.3%‐46.1%) (n = 3)	55.8% (35.5%‐74.4%) (n = 3)	a	a	5.2% (3.5%‐7.6%) (n = 4)	15.0% (6.3%‐31.5%) (n = 4)
Mutant allele‐specific PCR	41.5% (30.0%‐53.9%) (n = 4)	51.2% (45.7%‐56.7%) (n = 4)	35.6% (26.0%‐46.5%) (n = 4)	44.7% (27.7%‐63.1%) (n = 4)	5.9% (4.5%‐7.7%) (n = 4)	22.5% (17.5%‐28.5%) (n = 3)
Next‐generation sequencing	16.5% (8.3%‐30.3%) (n = 3)	a	a	a	4.2% (1.9%‐9.3%) (n = 4)	17.8% (12.6%‐24.6%) (n = 4)
Not reported	43.6% (35.4%‐52.2%) (n = 5)	49.5% (42.3%‐56.7%) (n = 4)	27.8% (19.7%‐37.7%) (n = 5)	39.8% (28.7%‐52.1%) (n = 4)	3.5% (1.7%‐7.1%) (n = 7)	12.3% (7.2%‐20.1%) (n = 7)
Other	37.4% (17.2%‐63.2%) (n = 4)	55.4% (25.2%‐82.1%) (n = 4)	31.9% (26.5%‐37.9%) (n = 4)	41.0% (36.1%‐6.1%) (n = 4)	4.7% (3.2%‐6.7%) (n = 3)	16.7% (8.8%‐29.3%) (n = 4)
Pyrosequencing	28.7% (16.9%‐44.3%) (n = 8)	31.6% (17.7%‐49.7%) (n = 8)	35.9% (27.6%‐45.3%) (n = 5)	44.9% (26.8%‐64.6%) (n = 5)	5.9% (4.2%‐8.1%) (n = 5)	16.7% (9.8%‐26.9%) (n = 5)
Sanger/direct sequencing (PCR)	30.7% (25.0%‐37.2%) (n = 18)	41.9% (33.6%‐50.7%) (n = 18)	42.1% (37.5%‐46.8%) (n = 15)	50.0% (44.2%‐55.9%) (n = 15)	4.7% (3.3%‐6.7%) (n = 10)	17.0% (13.4%‐21.4%) (n = 9)
Median length of follow‐up						
<3 y	28.2% (21.8%‐35.5%) (n = 7)	38.0% (21.6%‐57.7%) (n = 7)	28.9% (21.7%‐37.4%) (n = 5)	44.9% (32.6%‐57.8%) (n = 5)	7.1% (4.2%‐11.6%) (n = 3)	24.0% (15.3%‐35.6%) (n = 3)
≥3 y	20.9% (13.0%‐31.8%) (n = 8)	28.0% (15.0%‐46.1%) (n = 8)	30.0% (24.5%‐36.1%) (n = 6)	42.7% (31.8%‐54.5%) (n = 6)	3.6% (2.3%‐5.6%) (n = 8)	9.6% (5.7%‐15.6%) (n = 8)
Cochran's *Q*	*P* = .243	*P* = .427	*P* = .837	*P* = .808	*P* = .053	***P* = .007**
STROBE score						
<16	41.8% (37.0%‐46.8%) (n = 11)	50.2% (43.5%‐56.8%) (n = 11)	40.6% (36.0%‐45.3%) (n = 10)	48.7% (41.9%‐55.5%) (n = 10)	3.7% (2.1%‐6.4%) (n = 6)	19.1% (14.7%‐24.3%) (n = 6)
≥16	28.9% (23.7%‐34.7%) (n = 27)	37.5% (29.1%‐46.7%) (n = 26)	33.85% (28.6%‐39.4%) (n = 20)	44.9% (39.5%‐50.5%) (n = 19)	4.6% (3.4%‐6.1%) (n = 21)	15.8% (12.2%‐20.1%) (n = 21)
Cochran's *Q*	***P* = .001**	***P* = .029**	*P* = .068	*P* = .405	*P* = .500	*P* = .293
Meta‐regression on STROBE score	−0.07 (−0.14 to 0.01) (n = 38)	−0.06 (−0.17 to 0.04) (n = 37)	−0.02 (−0.08 to 0.05) (n = 30)	−0.00 (−0.07 to 0.07) (n = 29)	0.03 (−0.16 to 0.22) (n = 27)	−0.07 (−0.25 to 0.11) (n = 27)

a Fewer than three studies available for subgroup analysis.

Bold values indicate associations reaching a statistical significance of *P* < 0.05.

As the definition of left‐ and right‐sided tumors varied across studies, sensitivity analyses were conducted using various definitions of tumor location. The summary prevalence of RAS and KRAS mutations among transverse colon tumors was 48.2% and 40.2%, respectively (Table S2). After excluding studies that did not specifically separate transverse colon tumors from right‐sided tumors, the prevalence of RAS mutations among right‐sided tumors increased from 41.3% to 48.2% while the prevalence of KRAS mutations decreased slightly from 46.3% to 45.4%. The difference in KRAS mutations by tumor location for this stratification was no longer statistically significant in these analyses. Sensitivity analyses were not conducted for BRAF mutations as only one study reported BRAF mutations among transverse colon tumors and right‐sided tumors excluding the transverse colon. Among studies that separated rectal tumors from left‐sided tumors, the summary prevalence of RAS, KRAS, and BRAF mutations among rectal tumors was 34.3%, 30.8%, and 8.8%, respectively (Table S3). After removing studies that did not specifically separate rectal tumors from left‐sided tumors, the prevalence among left‐sided colon tumors did not change substantially from the original analyses (RAS: 36.4% vs 32.4%, KRAS: 34.7% vs 35.8%, BRAF: 4.4% vs 4.3%). The prevalence of RAS mutations by tumor location was no longer statistically significant in these analyses.

No visual or statistical evidence of publication bias was present in the RAS and KRAS prevalence analyses for either right‐ or left‐sided tumors. However, visual asymmetry was observed in the BRAF prevalence analyses for both tumor sides with more studies scattered about the left side of the mean, indicating that the estimate may be skewed toward zero (data not shown). Egger's regression method was statistically significant for publication bias in both left‐ and right‐sided tumors. The Duval and Tweedie trim and fill method was used to impute missing studies to the right of the mean for both analyses, but the estimates using the imputed studies were both similar to (within 10% of) the original estimates (left side: 4.6% vs original 4.3%; right side: 16.8% vs original 16.3%).

Among the included studies, only three provided data on survival outcomes specific to both tumor sidedness and mutation status. BRAF mutant tumors had poorer median OS than wild‐type among both left‐ and right‐sided tumors in two studies.^19^, ^31^ Also, KRAS mutant tumors had poorer OS than wild‐type tumors among left‐sided tumors, but improved OS and PFS compared to wild‐type among right‐sided tumors.^31^, ^67^ However, several studies evaluated the prognostic impact of tumor sidedness regardless of mutation status.^6^, ^7^, ^49^, ^58^, ^66^, ^67^, ^68^, ^70^ In exploratory meta‐analyses summarizing the results of these studies, right‐sided mCRC tumors had significantly decreased OS (HR: 1.64, 95% CI: 1.43‐1.88) and PFS (HR: 1.33, 95% CI: 1.15‐1.55) compared to left‐sided tumors.

## 4 DISCUSSION

This systematic review and meta‐analysis of mutation prevalence by tumor location among mCRC patients identified a significant difference in prevalence of both KRAS and BRAF mutations by tumor location, with mutations more frequent among right‐sided colon cancers than left‐sided tumors. Although there was statistically significant publication bias for the BRAF prevalence analyses, the addition of hypothetical “missing” studies did not significantly change the estimate of BRAF prevalence in right‐ or left‐sided colon cancers. Several potential sources of heterogeneity in mutation prevalence by tumor side were identified, including study design and study country, which may be a result of potential selection bias in the included studies. To our knowledge, this is the first meta‐analysis of KRAS and BRAF prevalence by tumor sidedness among patients with mCRC.

Our finding of a higher prevalence of BRAF mutations in right‐sided tumors is consistent with a recent meta‐analysis that reported a significant association between right‐sided colon cancer and BRAF V600E mutation (odds ratio: 4.85; 95% CI: 3.59‐6.56).^71^ Clinical trials have reported BRAF mutations were not predictive of response to cetuximab^72^ or panitumumab,^73^ although the sample sizes were small. The PICCOLO study identified an increased risk of mortality for BRAF‐mutated patients undergoing treatment with irinotecan and panitumumab compared with irinotecan alone.^73^ Further research is needed to evaluate predictive significance of BRAF mutation status in anti‐EGFR therapy.

Recent research has emphasized the difference in the epidemiologic, clinical, and molecular characteristics of right‐sided and left‐sided CRC, and they are generally considered distinct diseases, as right‐sided tumors are known to have worse prognosis than left‐sided tumors.^74^, ^75^, ^76^ However, in a pooled analysis of six RCTs, patients with right‐sided tumors continued to have a worse prognosis compared to left‐sided tumors even among RAS wild‐type mCRC patients, suggesting that the difference in mutation frequencies by tumor side is unlikely the sole factor accounting for the prognostic difference between left‐sided and right‐sided tumors.^75^ While the National Comprehensive Cancer Network (NCCN) guidelines recommend anti‐EGFR therapy in KRAS/NRAS/BRAF wild‐type and left‐sided mCRC only,^77^ no specific treatment recommendations are provided for KRAS/NRAS/BRAF mutant or right‐sided mCRC. As mCRC treatment continues to move in the direction of targeted approaches and personalized medicine, additional research on the impact of mutation status by tumor location could provide more options for these patients.

The strengths of this study include the breadth of literature that was searched to identify all studies of mCRC patients reporting both tumor sidedness and mutation status published within the last decade. Moreover, we contacted the authors of 65 studies of CRC patients that evaluated both variables but either did not present tumor sidedness by mutation status or did not report results for metastatic patients specifically. Although we only received responses from five authors, we were able to include additional data for these studies not reported in the published literature. There was statistically significant publication bias in the BRAF prevalence analyses for both left‐ and right‐sided tumors; however, the addition of hypothetical “missing” studies due to publication bias using Duval and Tweedie's method resulted in minimal changes from the original estimates, indicating that the estimated prevalence of BRAF mutations by tumor sidedness should not be substantially impacted by publication bias. Finally, few studies reported overall or PFS by both tumor sidedness and mutation status. As both variables have been associated with prognostic impact,^13^, ^14^, ^16^, ^78^ the combination of mutation status by tumor side should be evaluated in future studies.

## 5 CONCLUSION

The prevalence of KRAS and BRAF mutations was found to vary significantly by tumor location, with mutations more prevalent among right‐sided metastatic colon cancers than left‐sided tumors. These results help to better characterize the mCRC patient population and may have implications for improved targeting of anti‐EGFR therapies. Further research is needed to evaluate survival differences by mutation status and primary tumor location combined.
