# Supplementary material for: Prevalence of RAS and BRAF mutations in metastatic colorectal cancer patients by tumor sidedness: A systematic review and meta‐analysis
Source: Cancer Med. 2019 Dec 19;9(3):1044–57. doi: 10.1002/cam4.2747 (PMC6997095; doi:10.1002/cam4.2747)
Supplement: Supplementary file 1 [file CAM4-9-1044-s001.docx]

Supplemental Table 1. Characteristics of included studies of RAS or BRAF mutation prevalence by tumor location in mCRC.

| **Author (Year)** | **Study design** | **Country** | **% Male** | **Mean age (Range)** | **N population tested with results** | **Mutation assessment method** | **Mutations assessed** | **STROBE score** | **Survival data by sidedness and mutation status** |
| --- | --- | --- | --- | --- | --- | --- | --- | --- | --- |
| Alonso-Espinaco (2014)^1^ | Retrospective observational | Spain | 62.7% | 61.7 (28-82) | 134 | Direct sequencing/PCR | KRAS, BRAF | 15 | No |
| Bae (2013)^2^ | Retrospective observational | Korea | 60.8% | 62 (20-90) | 115 | NR | KRAS, BRAF | 17 | OS, PFS |
| Boeckx (2017)^3^ | Retrospective analysis of an RCT (PRIME) | Multi-country | 64.7% | 62 (24-82) | 204 | NR | BRAF | 16 | No |
|  | Retrospective analysis of an RCT (PEAK) | Multi-country | 67.8% | 62 (23-82) | 68 | NR | BRAF |  |  |
| Brulé (2015)^4^ | Retrospective analysis of an RCT | Multi-country | 60.9% | 63.5 (30.7-82.6) | 171 | NR | KRAS, BRAF | 17 | No |
| Cercek (2017)^5^ | Retrospective observational | USA | 54.0% | 58.3 (NR) | 982 | Sanger sequencing or Sequenom mass-spec | RAS, KRAS, NRAS | 20 | No |
| Cremolini (2015)^6^ | Retrospective observational | Italy | 59% | 65 (28-86) | 629 | Direct sequencing or Sequenom mass-spec | BRAF | 16 | No |
| Gorukmez (2016)^7^ | Retrospective observational | Turkey | 58% | 57 (30-70) | 50 | BigDye Terminator v3.1 | KRAS | 14 | No |
| Harlé (2015)^8^ | Retrospective observational | France | 57.63% | 69.2 ±10.7 | 177 | PCR/Pyrosequencing | RAS | 12 | No |
| Ilie (2014)^9^ | Retrospective observational | France | 68% | 67.8 (52-86) | 489 | Pyrosequencing or Sanger sequencing | KRAS, BRAF | 15 | No |
| Karagkounis (2013)^10^ | Prospective observational | USA | 66% | 61 (33-84) | 202 | Direct sequencing | KRAS | 19 | No |
| Kawazoe (2015)^11^ | Retrospective observational | Japan | 62.9% | 64 (38-82) | 264 | Luminex (xMAP) technology | KRAS, BRAF | 18 | No |
| Kemeny (2014)^12^ | Retrospective observational | USA | 58.6% | 56 (36-79) | 169 | PCR/iPLEX Assay | KRAS | 17 | No |
| Kodaz (2015)^13^ | Retrospective observational | Turkey | 64.6% | 61 (27-81) | 189 | Therascreen® KRAS Pyro Kit | KRAS | 14 | No |
| Korphaisarn (2015)^14^ | Retrospective observational | Thailand | 50% | 63 (33-95) | 29 | AS-PCR | BRAF | 17 | No |
| Kuramochi (2016)^15^ | Retrospective observational | Japan | 67.31% | 61.5 (32-91) | 49 | Direct sequencing | KRAS | 15 | No |
| Loupakis (2015)^16^ | Prospective observational | Multi-country | 63% | NR (≤50: 15%) | 200 | NR | BRAF | 18 | No |
| Michl (2015)^17^ | Retrospective observational | Germany | 58.5% | NR (32-94) | 65 | Pyrosequencing | KRAS, BRAF | 18 | No |
| Morris (2014)^18^ | Retrospective observational | USA | 57% | 54.6 | 473 | PCR | KRAS, BRAF | 18 | No |
| Nitsche (2016)^19^ | Prospective observational | Germany | 56.48% | Right: 68  Left: 64  (20-98) | 8 | NR | KRAS, BRAF | 18 | No |
| Ostrizkova (2016)^20^ | Retrospective observational | Czech Republic | NR | NR | 562 | NR | KRAS | 9 | No |
| Osumi (2016)^21^ | Prospective observational | Japan | 53% | 63 ± 10.4 | 132 | Luminex | RAS | 16 | No |
| Pai (2012)^22^ | Retrospective observational | USA | NR | NR | 34 | BRAF: real-time PCR  KRAS: BigDye Terminator v3.1 | KRAS, BRAF | 13 | No |
| Pereira (2015)^23^ | Retrospective observational | USA | 58.9% | 55 (25-85) | 494 | Sanger sequencing or Sequenom mass-spec | KRAS | 17 | No |
| Price (2011)^24^ | Phase III RCT | Australia | 63% | 69 (36-85) | 315 | High resolution melting point PCR | KRAS, BRAF | 16 | No |
| Price (2014)^25^ | Retrospective observational | Australia | 48.7% | 75.8 | 2877 | NR | KRAS | 8 | No |
| Price (2016)^26^ | Retrospective observational | Australia | 59.47% | 71.5 (30.2 – 89.7) | 227 | NR | BRAF | 14 | No |
| Roma (2016)^27^ | Retrospective observational | Italy | 57.9% | NR | 840 | PCR with BigDye Terminator v3.1 | BRAF | 14 | No |
| Saito (2014)^28^ | Prospective observational | Japan | 57.8% | 63.5 (20-82) | 109 | ARMS-Scorpion Assay and Direct Sequencing | KRAS | 16 | No |
| Schafroth (2015)^29^ | Retrospective observational | Germany | 53.7% | Negative: 64.3  Positive: 69.4 | 65 | VE1 Immunohistochemistry | BRAF | 16 | No |
| Schirripa (2015)^30^ | Prospective observational | Italy | 59.1% | 64 (25-88) | 786 | BigDye Terminator v3.1 | KRAS, BRAF, NRAS | 17 | No |
| Schweiger (2014)^31^ | Prospective observational | Austria | 51.3% | 64 (37-79) | 39 | AmpliTaqGold PCR Master Mix | KRAS | 20 | No |
| Selcukbiricik (2013)^32^ | Retrospective observational | Turkey | 37.2% | 60.5 (27-83) | 172 | Real-time PCR and Qia Gen Kit | KRAS | 13 | No |
| Soeda (2014)^33^ | Retrospective observational | Japan | 57.22% | 65 (37-81) | 194 | CEQ2000XL DNA Analysis System | KRAS | 16 | No |
| Soeda (2014)^34^ | Phase II RCT | Japan | 62.8% | 67 (50-81) | 43 | PCR | KRAS | 15 | No |
| Teng (2012)^35^ | Retrospective observational | Taiwan | 60.6% | 61 | 292 | BigDye Terminator v3.1 | KRAS, BRAF | 18 | No |
| Tran (2011)^36^ | Retrospective observational | USA, Australia | 55.2% | 66 | 524 | Mutation-specific real-time PCR | BRAF | 17 | No |
| Ulivi (2017)^37^ | Retrospective analysis of an RCT | Italy | 57.4% | Right-sided: 68 (34-83)  Left-sided: 63 (34-83) | 122 | Pyrosequencing | KRAS, BRAF, NRAS | 17 | No |
| Umeda (2013)^38^ | Prospective observational | Japan | 55% | 64 ± 6.7 | 100 | Directing sequencing/PCR | KRAS, BRAF | 16 | No |
| Von Einem (2014)^39^ | Phase II RCT | Germany | 71.9% | Left: 63  Right: 61 (32-77) | 146 | NR | KRAS, BRAF | 14 | OS, PFS |
| Voutsina (2013)^40^ | Retrospective observational | Greece | 51.8% | NR | 83 | Real time PCR, Sanger sequencing, TaqMan SNP Genotyping Assays | KRAS, BRAF | 15 | No |
| Yaeger (2014)^41^ | Retrospective observational | USA | 52% | 64 (28-86) | 1941 | Sequenom assay or Sanger sequencing | BRAF | 18 | OS |
| Yaeger (2015)^42^ | Retrospective observational | USA | 53.7% | 58 (20-91) | 1095 | Sanger sequencing (pre-10/2010), Sequenom mass-spec | RAS | 18 | No |
| Yamashita (2017)^43^ | Retrospective observational | USA | 64.9% | Right-sided: 57 (42-78)  Left-sided: 59 (28-92) | 74 | NR | RAS | 19 | No |
| Yoon (2015)^44^ | Retrospective observational | Korea | 66.7% | NR | 48 | NR | KRAS, BRAF | 16 | No |

NR, Not Reported; OS, Overall Survival; PFS, Progression-free Survival; RCT, Randomized controlled trial; STROBE, Strengthening the Reporting of Observational Studies in Epidemiology.

Supplemental Table 2. Prevalence of RAS and KRAS mutations by primary tumor location, including transverse colon tumors

|  | **RAS mutation** | | | **KRAS mutation** | | |
| --- | --- | --- | --- | --- | --- | --- |
|  | **Left-sided tumors** | **Right-sided tumors*** | **Transverse colon tumors** | **Left-sided tumors** | **Right-sided tumors*** | **Transverse colon tumors** |
| Mutation prevalence (95% CI) | 32.4%  (28.4 – 36.7%) | 48.2% (39.3-57.3%) | 39.8% (29.3-51.3%) | 35.8%  (32.2 – 39.6%) | 45.4% (36.7-54.3%) | 40.2% (29.5-51.9%) |
| N studies included | 38 | 7 | 7 | 30 | 6 | 6 |
| P-value for heterogeneity; I^2^ value | <0.0001; 95.4% | <0.0001; 90.4% | 0.941; 0.0% | <0.0001; 91.0% | <0.001; 88.3% | 0.907; 0.0% |
| Cochran’s Q test for heterogeneity between groups | 0.005 | | | 0.125 | | |

*Right-sided tumors, excluding tumors in the transverse colon

Supplemental Table 3. Prevalence of RAS, KRAS, and BRAF mutations by primary tumor location, including rectal tumors

|  | **RAS mutation** | | | **KRAS mutation** | | | **BRAF mutation** | | |
| --- | --- | --- | --- | --- | --- | --- | --- | --- | --- |
|  | **Left-sided colon tumors*** | **Rectal tumors** | **Right-sided colon tumors** | **Left-sided colon tumors*** | **Rectal tumors** | **Right-sided colon tumors** | **Left-sided colon tumors*** | **Rectal tumors** | **Right-sided colon tumors** |
| Mutation prevalence (95% CI) | 36.4% (30.7-42.6%) | 34.3% (27.4-41.9%) | 41.3%  (35.4 – 47.5%) | 34.7% (28.0-42.1%) | 30.8% (24.6-37.8%) | 46.3%  (42.3 – 50.4%) | 4.4% (3.1-6.2%) | 8.8% (3.6-20.2%) | 16.3%  (13.5 – 19.6%) |
| N studies included | 14 | 14 | 37 | 12 | 12 | 29 | 7 | 6 | 27 |
| P-value for heterogeneity; I^2^ value | <0.001; 93.6% | <0.0001; 84.5% | <0.0001; 97.4% | <0.0001; 93.7% | <0.0001; 78.6% | <0.0001; 91.7% | 0.005; 68.1% | <0.0001; 80.4% | <0.0001; 91.7% |
| Cochran’s Q test for heterogeneity between groups | 0.312 | | | <0.0001 | | | <0.0001 | | |

*Left-sided colon tumors, excluding rectal tumors

References

1. Alonso-Espinaco V, Cuatrecasas M, Alonso V, Escudero P, Marmol M, Horndler C, Ortego J, Gallego R, Codony-Servat J, Garcia-Albeniz X, Jares P, Castells A, et al. RAC1b overexpression correlates with poor prognosis in KRAS/BRAF WT metastatic colorectal cancer patients treated with first-line FOLFOX/XELOX chemotherapy. *European journal of cancer (Oxford, England : 1990)* 2014;**50**: 1973-81.

2. Bae JM, Kim JH, Cho NY, Kim TY, Kang GH. Prognostic implication of the CpG island methylator phenotype in colorectal cancers depends on tumour location. *British journal of cancer* 2013;**109**: 1004-12.

3. Boeckx N, Koukakis R, Op de Beeck K, Rolfo C, Van Camp G, Siena S, Tabernero J, Douillard JY, Andre T, Peeters M. Primary tumor sidedness has an impact on prognosis and treatment outcome in metastatic colorectal cancer: results from two randomized first-line panitumumab studies. *Annals of oncology : official journal of the European Society for Medical Oncology* 2017;**28**: 1862-8.

4. Brule SY, Jonker DJ, Karapetis CS, O'Callaghan CJ, Moore MJ, Wong R, Tebbutt NC, Underhill C, Yip D, Zalcberg JR, Tu D, Goodwin RA. Location of colon cancer (right-sided versus left-sided) as a prognostic factor and a predictor of benefit from cetuximab in NCIC CO.17. *European journal of cancer (Oxford, England : 1990)* 2015;**51**: 1405-14.

5. Cercek A, Braghiroli MI, Chou JF, Hechtman JF, Kemeny N, Saltz L, Capanu M, Yaeger R. Clinical Features and Outcomes of Patients with Colorectal Cancers Harboring NRAS Mutations. *Clinical cancer research : an official journal of the American Association for Cancer Research* 2017;**23**: 4753-60.

6. Cremolini C, Di Bartolomeo M, Amatu A, Antoniotti C, Moretto R, Berenato R, Perrone F, Tamborini E, Aprile G, Lonardi S, Sartore-Bianchi A, Fontanini G, et al. BRAF codons 594 and 596 mutations identify a new molecular subtype of metastatic colorectal cancer at favorable prognosis. *Annals of oncology : official journal of the European Society for Medical Oncology* 2015;**26**: 2092-7.

7. Gorukmez O, Yakut T, Gorukmez O, Ozemri Sag S, Karkucak M, Kanat O. Distribution of KRAS and BRAF Mutations in Metastatic Colorectal Cancers in Turkish Patients. *Asian Pac J Cancer Prev* 2016;**17**: 1175-9.

8. Harle A, Filhine-Tresarrieu P, Husson M, Boidot R, Rouyer M, Dubois C, Leroux A, Merlin JL. Rare RAS Mutations in Metastatic Colorectal Cancer Detected During Routine RAS Genotyping Using Next Generation Sequencing. *Targeted oncology* 2016;**11**: 363-70.

9. Ilie MI, Long-Mira E, Hofman V, Mouroux J, Vignaud JM, Gauchotte G, Begueret H, Merlio JP, Emile JF, Hebuterne X, Hofman P. BRAFV600E mutation analysis by immunohistochemistry in patients with thoracic metastases from colorectal cancer. *Pathology* 2014;**46**: 311-5.

10. Karagkounis G, Torbenson MS, Daniel HD, Azad NS, Diaz LA, Jr., Donehower RC, Hirose K, Ahuja N, Pawlik TM, Choti MA. Incidence and prognostic impact of KRAS and BRAF mutation in patients undergoing liver surgery for colorectal metastases. *Cancer* 2013;**119**: 4137-44.

11. Kawazoe A, Shitara K, Fukuoka S, Kuboki Y, Bando H, Okamoto W, Kojima T, Fuse N, Yamanaka T, Doi T, Ohtsu A, Yoshino T. A retrospective observational study of clinicopathological features of KRAS, NRAS, BRAF and PIK3CA mutations in Japanese patients with metastatic colorectal cancer. *BMC Cancer* 2015;**15**: 258.

12. Kemeny NE, Chou JF, Capanu M, Gewirtz AN, Cercek A, Kingham TP, Jarnagin WR, Fong YC, DeMatteo RP, Allen PJ, Shia J, Ang C, et al. KRAS mutation influences recurrence patterns in patients undergoing hepatic resection of colorectal metastases. *Cancer* 2014;**120**: 3965-71.

13. Kodaz H, Hacibekiroglu I, Erdogan B, Turkmen E, Tozkir H, Albayrak D, Uzunoglu S, Cicin I. Association between specific KRAS mutations and the clinicopathological characteristics of colorectal tumors. *Molecular and clinical oncology* 2015;**3**: 179-84.

14. Korphaisarn K, Pongpaibul A, Limwongse C, Roothumnong E, Klaisuban W, Nimmannit A, Jinawath A, Akewanlop C. Deficient DNA mismatch repair is associated with favorable prognosis in Thai patients with sporadic colorectal cancer. *World journal of gastroenterology* 2015;**21**: 926-34.

15. Kuramochi H, Nakamura A, Nakajima G, Kaneko Y, Araida T, Yamamoto M, Hayashi K. PTEN mRNA expression is less pronounced in left- than right-sided colon cancer: a retrospective observational study. *BMC Cancer* 2016;**16**: 1-8.

16. Loupakis F, Yang D, Yau L, Feng S, Cremolini C, Zhang W, Maus MK, Antoniotti C, Langer C, Scherer SJ, Muller T, Hurwitz HI, et al. Primary tumor location as a prognostic factor in metastatic colorectal cancer. *Journal of the National Cancer Institute* 2015;**107**.

17. Michl M, Heinemann V, Jung A, Engel J, Kirchner T, Neumann J. Expression of cancer stem cell markers in metastatic colorectal cancer correlates with liver metastasis, but not with metastasis to the central nervous system. *Pathology, research and practice* 2015;**211**: 601-9.

18. Morris VK, Lucas FA, Overman MJ, Eng C, Morelli MP, Jiang ZQ, Luthra R, Meric-Bernstam F, Maru D, Scheet P, Kopetz S, Vilar E. Clinicopathologic characteristics and gene expression analyses of non-KRAS 12/13, RAS-mutated metastatic colorectal cancer. *Annals of oncology : official journal of the European Society for Medical Oncology* 2014;**25**: 2008-14.

19. Nitsche U, Stogbauer F, Spath C, Haller B, Wilhelm D, Friess H, Bader FG. Right Sided Colon Cancer as a Distinct Histopathological Subtype with Reduced Prognosis. *Digestive surgery* 2016;**33**: 157-63.

20. Ostrizkova L, Petruželka L, Hejduk K, Zdražilová-Dubská L, Vocka M, Brancikova D, Bencsiková B, Vyzula R, Obermannová R. poster discussions 2016.

21. Osumi H, Shinozaki E, Suenaga M, Matsusaka S, Konishi T, Akiyoshi T, Fujimoto Y, Nagayama S, Fukunaga Y, Ueno M, Mise Y, Ishizawa T, et al. RAS mutation is a prognostic biomarker in colorectal cancer patients with metastasectomy. *International journal of cancer* 2016;**139**: 803-11.

22. Pai RK, Jayachandran P, Koong AC, Chang DT, Kwok S, Ma L, Arber DA, Balise RR, Tubbs RR, Shadrach B, Pai RK. BRAF-mutated, microsatellite-stable adenocarcinoma of the proximal colon: an aggressive adenocarcinoma with poor survival, mucinous differentiation, and adverse morphologic features. *The American journal of surgical pathology* 2012;**36**: 744-52.

23. Pereira AA, Rego JF, Morris V, Overman MJ, Eng C, Garrett CR, Boutin AT, Ferrarotto R, Lee M, Jiang ZQ, Hoff PM, Vauthey JN, et al. Association between KRAS mutation and lung metastasis in advanced colorectal cancer. *British journal of cancer* 2015;**112**: 424-8.

24. Price TJ, Hardingham JE, Lee CK, Weickhardt A, Townsend AR, Wrin JW, Chua A, Shivasami A, Cummins MM, Murone C, Tebbutt NC. Impact of KRAS and BRAF Gene Mutation Status on Outcomes From the Phase III AGITG MAX Trial of Capecitabine Alone or in Combination With Bevacizumab and Mitomycin in Advanced Colorectal Cancer. *Journal of clinical oncology : official journal of the American Society of Clinical Oncology* 2011;**29**: 2675-82.

25. Price TJ, Beeke C, Padbury R, Maddern G, Roder D, Moore J, Townsend AR, Roy A, Hocking C, Karapetis CS. Right (R) or left (L) primary site of colorectal cancer and outcomes for metastatic colorectal cancer (mCRC): Results from the south Australian registry of mCRC: American Society of Clinical Oncology, 2014.

26. Price TJ, Beeke C, Townsend AR, Lo L, Amitesh R, Padbury R, Roder D, Maddern G, Moore J, Karapetis C. BRAF Mutation Testing and Metastatic Colorectal Cancer in the Community Setting: Is There an Urgent Need for More Education? *Molecular diagnosis & therapy* 2016;**20**: 75-82.

27. Roma C, Rachiglio AM, Pasquale R, Fenizia F, Iannaccone A, Tatangelo F, Antinolfi G, Parrella P, Graziano P, Sabatino L, Colantuoni V, Botti G, et al. BRAF V600E mutation in metastatic colorectal cancer: Methods of detection and correlation with clinical and pathologic features. *Cancer biology & therapy* 2016;**17**: 840-8.

28. Saito N, Tomita S, Ichikawa K, Mitomi H, Imura J, Fujimori T. Analysis of KRAS mutations in cases of metastatic colorectal cancer at a single institution in Tochigi, Japan. *Pathobiology : journal of immunopathology, molecular and cellular biology* 2014;**81**: 133-7.

29. Schafroth C, Galvan JA, Centeno I, Koelzer VH, Dawson HE, Sokol L, Rieger G, Berger MD, Hadrich M, Rosenberg R, Nitsche U, Schnuriger B, et al. VE1 immunohistochemistry predicts BRAF V600E mutation status and clinical outcome in colorectal cancer. *Oncotarget* 2015;**6**: 41453-63.

30. Schirripa M, Cremolini C, Loupakis F, Morvillo M, Bergamo F, Zoratto F, Salvatore L, Antoniotti C, Marmorino F, Sensi E, Lupi C, Fontanini G, et al. Role of NRAS mutations as prognostic and predictive markers in metastatic colorectal cancer. *International journal of cancer* 2015;**136**: 83-90.

31. Schweiger T, Hegedus B, Nikolowsky C, Hegedus Z, Szirtes I, Mair R, Birner P, Dome B, Lang G, Klepetko W, Ankersmit HJ, Hoetzenecker K. EGFR, BRAF and KRAS status in patients undergoing pulmonary metastasectomy from primary colorectal carcinoma: a prospective follow-up study. *Annals of surgical oncology* 2014;**21**: 946-54.

32. Selcukbiricik F, Erdamar S, Ozkurt CU, Molinas Mandel N, Demirelli F, Ozguroglu M, Tural D, Buyukunal E, Serdengecti S. The role of K-RAS and B-RAF mutations as biomarkers in metastatic colorectal cancer. *Journal of BUON : official journal of the Balkan Union of Oncology* 2013;**18**: 116-23.

33. Soeda H, Shimodaira H, Watanabe M, Suzuki T, Gamo M, Takahashi M, Komine K, Kato S, Ishioka C. KRAS mutation in patients with metastatic colorectal cancer does not preclude benefit from oxaliplatin-or irinotecan-based treatment. *Molecular and clinical oncology* 2014;**2**: 356-62.

34. Soeda H, Shimodaira H, Gamoh M, Ando H, Isobe H, Suto T, Takahashi S, Kakudo Y, Amagai K, Mori T, Watanabe M, Yamaguchi T, et al. Phase II trial of cetuximab plus irinotecan for oxaliplatin- and irinotecan-based chemotherapy-refractory patients with advanced and/or metastatic colorectal cancer: evaluation of efficacy and safety based on KRAS mutation status (T-CORE0801). *Oncology* 2014;**87**: 7-20.

35. Teng HW, Huang YC, Lin JK, Chen WS, Lin TC, Jiang JK, Yen CC, Li AF, Wang HW, Chang SC, Lan YT, Lin CC, et al. BRAF mutation is a prognostic biomarker for colorectal liver metastasectomy. *Journal of surgical oncology* 2012;**106**: 123-9.

36. Tran B, Kopetz S, Tie J, Gibbs P, Jiang ZQ, Lieu CH, Agarwal A, Maru DM, Sieber O, Desai J. Impact of BRAF mutation and microsatellite instability on the pattern of metastatic spread and prognosis in metastatic colorectal cancer. *Cancer* 2011;**117**: 4623-32.

37. Ulivi P, Scarpi E, Chiadini E, Marisi G, Valgiusti M, Capelli L, Casadei Gardini A, Monti M, Ruscelli S, Frassineti GL, Calistri D, Amadori D, et al. Right- vs. Left-Sided Metastatic Colorectal Cancer: Differences in Tumor Biology and Bevacizumab Efficacy. *International journal of molecular sciences* 2017;**18**.

38. Umeda Y, Nagasaka T, Mori Y, Sadamori H, Sun DS, Shinoura S, Yoshida R, Satoh D, Nobuoka D, Utsumi M, Yoshida K, Yagi T, et al. Poor prognosis of KRAS or BRAF mutant colorectal liver metastasis without microsatellite instability. *Journal of hepato-biliary-pancreatic sciences* 2013;**20**: 223-33.

39. von Einem JC, Heinemann V, von Weikersthal LF, Vehling-Kaiser U, Stauch M, Hass HG, Decker T, Klein S, Held S, Jung A, Kirchner T, Haas M, et al. Left-sided primary tumors are associated with favorable prognosis in patients with KRAS codon 12/13 wild-type metastatic colorectal cancer treated with cetuximab plus chemotherapy: an analysis of the AIO KRK-0104 trial. *Journal of cancer research and clinical oncology* 2014;**140**: 1607-14.

40. Voutsina A, Tzardi M, Kalikaki A, Zafeiriou Z, Papadimitraki E, Papadakis M, Mavroudis D, Georgoulias V. Combined analysis of KRAS and PIK3CA mutations, MET and PTEN expression in primary tumors and corresponding metastases in colorectal cancer. *Modern pathology : an official journal of the United States and Canadian Academy of Pathology, Inc* 2013;**26**: 302-13.

41. Yaeger R, Cercek A, Chou JF, Sylvester BE, Kemeny NE, Hechtman JF, Ladanyi M, Rosen N, Weiser MR, Capanu M, Solit DB, D'Angelica MI, et al. BRAF mutation predicts for poor outcomes after metastasectomy in patients with metastatic colorectal cancer. *Cancer* 2014;**120**: 2316-24.

42. Yaeger R, Cowell E, Chou JF, Gewirtz AN, Borsu L, Vakiani E, Solit DB, Rosen N, Capanu M, Ladanyi M, Kemeny N. RAS mutations affect pattern of metastatic spread and increase propensity for brain metastasis in colorectal cancer. *Cancer* 2015;**121**: 1195-203.

43. Yamashita S, Odisio BC, Huang SY, Kopetz SE, Ahrar K, Chun YS, Conrad C, Aloia TA, Gupta S, Harmoush S, Hicks ME, Vauthey JN. Embryonic origin of primary colon cancer predicts survival in patients undergoing ablation for colorectal liver metastases. *European journal of surgical oncology : the journal of the European Society of Surgical Oncology and the British Association of Surgical Oncology* 2017;**43**: 1040-9.

44. Yoon YS, Kim J, Hong SM, Lee JL, Kim CW, Park IJ, Lim SB, Yu CS, Kim JC. Clinical implications of mucinous components correlated with microsatellite instability in patients with colorectal cancer. *Colorectal disease : the official journal of the Association of Coloproctology of Great Britain and Ireland* 2015;**17**: O161-7.
